# A nanoparticulate pre-chemosensitizer for efficacious chemotherapy of multidrug resistant breast cancer

**DOI:** 10.1038/srep21459

**Published:** 2016-02-15

**Authors:** Shengrong Guo, Li Lv, Yuanyuan Shen, Zhongliang Hu, Qianjun He, Xiaoyuan Chen

**Affiliations:** 1 School of Pharmacy, Shanghai Jiao Tong University, Shanghai 200240, China; 2 School of Chemical and Process Engineering, University of Leeds, Leeds LS3 9BD, UK; 3 Guangdong Key Laboratory for Biomedical Measurements and Ultrasound Imaging, Department of Biomedical Engineering, School of Medicine, Shenzhen University, Shenzhen, Guangdong, China; 4 Laboratory of Molecular Imaging and Nanomedicine (LOMIN), National Institute of Biomedical Imaging and Bioengineering (NIBIB), National Institutes of Health (NIH), Bethesda MD 20892, USA

## Abstract

Small-molecule chemosensitizers can reverse cancer multidrug resistance (MDR), thus significantly improving the *in vitro* effect of chemotherapy drugs for MDR cancer cells, however, their *in vivo* effects are not always very good, because they are difficult to effectively accumulate in tumor and enter the same cancer with chemotherapy drugs after systemic administration due to individual biopharmaceutical properties. To overcome these limitations, here we study a novel nanoparticular pre-chemosensitizer which can be also used as nanocarrier of chemotherapy drugs. We take an ‘all in one’ approach to develop a self-assembled nanoparticle formula of amphiphilic poly(curcumin-dithiodipropionic acid)-b-poly(ethylene glycol)-biotin. The nanoparticle is capable of tumor-targeted delivery, responsive degradation at the intracellular level of glutathione and subsequent intracellular co-release of the chemosensitizer curcumin and the encapsulated chemotherapeutic drug doxorubicin to maximize a synergistic effect of chemosensitization and chemotherapy. We demonstrate that the antitumor efficacy of nanoparticle is much superior to that of doxorubicin in the multidrug resistant MCF-7/ADR xenografted nude mice.

Chemotherapy is the most common treatment modality for cancer clinically. Unfortunately, its antitumor effects are not so good as expected, especially for multidrug resistance (MDR) cancer, moreover its adverse effects are always great. Thus, it is urgent and necessary to develop a novel approach to effectively improve chemtherapy efficacy and reduce toxicity. As is well-known, most of chemotherapy drugs have a very narrow therapeutic index/window, frequently show toxicity to healthy tissues or organs even at dosages lower than required for a therapeutic effect. Moreover, multiple dose administrations of drugs often induce cancer MDR, which, in turn, would greatly compromise chemotherapeutic effects^1^,^2^,^3^,^4^. Therefore, it would not be very optimistic to only rely on the development of more potent chemotherapeutic agents for the treatment of MDR cancer. Actually, once MDR occurs, which is almost inevitable in clinical practice, the effective drug dose will have to be remarkably enhanced by 1–2 orders of magnitude as compared to that for the original drug-sensitive cancer, and therefore leads to unbearable systemic toxicity for cancer patients. In this case, it is essential to reverse MDR and enhance drug sensibility. Meanwhile, it is beneficial to facilitate drug accumulation in tumor and reduce drug distribution in healthy tissues so as to allow drugs as many as possible to act on cancer. Chemosensitizer are usually small-molecule compounds that can reverse MDR and make tumor cells more sensitive to chemotherapeutic agents, thus improving chemotherapy efficacy. However, their *in vivo* effects are not always very good, because they are difficult to effectively accumulate in tumor and enter the same cancer with chemotherapy drugs after systemic administration.

Up to now, a variety of nano-sized drug delivery systems (NanoDDS) have been developed for passive tumor delivery via the enhanced permeability and retention (EPR) effect and for active delivery by the virtue of specific biological characteristics of tumor (e.g., overexpressed receptors) to increase the distribution of drugs in cancer tissue/cells and reduce their toxicity towards normal tissues^5^,^6^,^7^,^8^,^9^,^10^,^11^,^12^,^13^,^14^. Drugs-incorporated NanoDDS can also bypass efflux pumps on the membrane to enhance endocytosis. However, many NanoDDS often have a low payload of drug and unexpected drug release profiles (burst release, too slow release, drug leakage during transport, etc.), which would reduce the amount of drugs that enter tumor cells, thus greatly decreasing their dual benefits of enhancing antitumor effects and reducing toxicity. Therefore, it is significant to realize both targeted delivery and responsive release of large amount of drugs by NanoDDS for the treatment of MDR cancer.

MDR cancer cells can facilitate the efflux of drugs through up-regulating efflux transporters (e.g. P-glycoprotein, etc.), which minimize intracellular drug amount^15^. In this case, high-dose therapy may enhance chemotherapeutic effect to a certain extent, but always causes severe systemic toxicity and associated negative outcomes in patients^16^,^17^. As mentioned above, the combined administration of chemosensitizer and chemotherapeutic drug should be able to improve the *in vivo* chemotherapy efficacy of MDR cancer in principle if these two agents can acumulate in cancer tissue/cells simultaneously and effectively. However, in fact, the factors affecting this cooperative effect are very complicated as individual biopharmaceutical and pharmacokinetic properties of different drug molecules make them difficult to diffuse into the same cancer cell at the same time after systemic drug administration. Therefore, the anti-MDR effect is often not satisfactory via the common route of systemic administration of chemotherapeutic agents and chemosensitizers. NanoDDS provides a good opportunity to co-deliver chemotherapeutic agents and chemosensitizers to MDR cancer cells. However, it remains a challenge to co-load high payloads of chemotherapeutic agents and chemosensitizers, especially hydrophobic ones, into a single nanoparticle, and also ensure no drug leakage during *in vivo* transportation and responsive co-release of drugs at the tumor site.

Herein, we developed a novel nanoparticular pre-chemosensitizer (as a precursor of small-molecule chemosensitizer), which can overcome the shortcomings of conventional small-molecule chemosensitizers and exerts its chemosensitization effect through loading chemotherapeutic agents, as well as co-delivering and co-releasing chemotherapeutic agents and chemosensitizers to the tumor cells. We used one self-assembled polycurcumin nanoparticle both as a pre-chemosensitizer and as a drug carrier to construct targeted NanoDDS for the effective treatment of MDR cancer *in vivo*. The nanoparticle is formed through self-assembly of amphiphilic poly(curcumin-dithiodipropionic acid)-b-poly(ethylene glycol)-biotin (PCDA-PEG-Biotin) copolymer, which consists of one hydrophilic outer shell of the PEG-Biotin block and one hydrophobic inner core of the PCDA block (Figure S1 and Fig. 1a). With such an unique structure, this nanoparticle is endowed with several desired functions/advantages: 1) extraordinarily high loading capacity of hydrophobic multi-drugs owing to the curcumin-polymerized hydrophobic core and the hydrophobic phase encapsulation of DOX; 2) stimuli-triggered intracellular multi-drug co-release through responsive degradation of nano-carrier at the intracellular level of glutathione (GSH); 3) cancer targeted multi-drug co-delivery by the EPR effect and the interaction of biotin and overexpressed biotin receptor on the surface of cancer cells; 4) stealth-shielding and long circulation owing to PEGylation. Notably, CUR, a hydrophobic diarylheptanoid, which is extracted from turmeric as food additive and traditional medicine for centuries in China and India, has proved to be a chemopreventive agent and also a chemo-/radio-sensitizer^18^,^19^,^20^. However, the *in vivo* application of CUR is limited due to its poor water solubility and poor stability under physiological condition or under UV/visible irradiation^21^,^22^. Unlike free CUR, the PCDA-PEG-Biotin is highly stable in water at physiological pH. This is because CUR is bonded to the PCDA backbone, and the assembled hydrophobic inner core protects CUR from rapid hydrolysis.

As shown in Fig. 1 and Figure S1, DOX is encapsulated in the hydrophobic inner core of the PCDA backbone to form the DOX@PCDA-PEG-Biotin formula. The hydrophilic outer PEG shell can offer protection against recognition and uptake by the reticuloendothelial system, allowing stealth-shielding and long circulation. After intravenous injection, the DOX@PCDA-PEG-Biotin NPs are expected to accumulate within the tumor tissue by the passive targeting effect (EPR effect)^23^, and then endocytosed into MCF-7/ADR cells by the biotin receptor-mediated active targeting effect^24^,^25^. The PCDA backbone would degrade at a significantly higher concentration of GSH in the cytosol than in the extracellular fluid, leading to the disassembly of the NPs and the co-release of DOX and CUR within the same cancer cell at the same time. The released CUR plays a role as a chemosensitizer to down-regulate the P-gp expression and also inhibit the ATP activity of MCF-7/ADR cells, suppressing the efflux of DOX and facilitating the intracellular accumulation of DOX. Therefore, the cytotoxicity of DOX against MCF-7/ADR cells is augmented significantly by the virtue of chemosensitization. In a word, this self-assembled polycurcumin nanoparticle can take full advantages of its ‘two-in-one’ combination of chemotherapeutic drug and pre-chemosensitizer, active and passive tumor targeting, and intracellular responsive multi-drug co-release properties to maximize the antitumor effect against MDR cancers.

## Results and Discussion

### Design, Preparation and Characterization of Biotin-PEG-PCDA and DOX@PCDA-PEG-Biotin NPs

**Figure 2:**
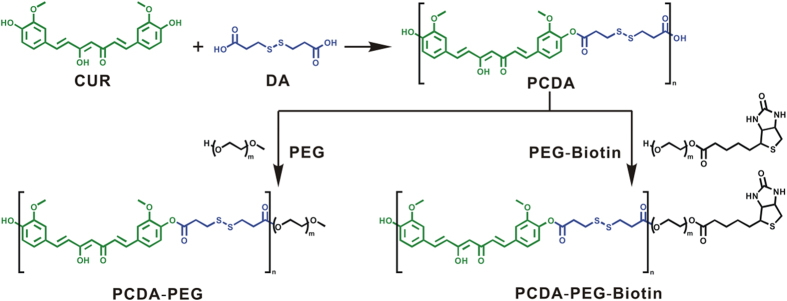
Synthesis routes of PCDA-PEG and PCDA-PEG-Biotin by a poly(active pharmaceutical ingredients) (PAPI) strategy.

In recent years, great efforts have been made to overcome CUR’s poor water solubility and stability^26^,^27^,^28^,^29^,^30^, including physical encapsulation of CUR in nanoparticles, chemical conjugation of CUR to hydrophilic compounds and the copolymerization of CUR with hydrophilic monomers, etc. For example, Shen group reported polycurcumin by condensation polymerization of curcumin with various hydrophilic PEGs^26^. Such polycondensates have the advantages of defined, high drug loading within the polymer backbones, leading to improved curcumin stability and tailored water-solubility. We designed and prepared Biotin-PEG-PCDA NPs through the use of a poly(active pharmaceutical ingredient) (PAPI) strategy where the API is incorporated into an intracellular cleavable polymer backbone in combination with the exploitation of self-assembly characteristics of amphiphilic di-block copolymers^28^. As shown in Fig. 2, PDCA was firstly synthesized via the co-polycondensation of CUR and dithiodipropionic acid (DA) at a molar ratio of 1:1. The synthesized PDCA was characterized by 1 H nuclear magnetic resonance (1 H-NMR) and Fourier transform infrared spectroscopy (FR-IR) techniques (Figures S2 and S4 in the Supporting Information). Furthermore, PEG or PEG-Biotin was conjugated at the end of PCDA by esterification at a molar ratio of 1:1. Wherein PEG-Biotin was obtained by esterification of biotin and PEG (Mw = 5,000 Dalton), possessing an average molecular weight of 6,000 Dalton.

The synthesized intermediate and final products were characterized by gel permeation chromatography (GPC), NMR and FT-IR (Figures S2–S4, Table S1 in the Supporting Information). The average molecular weights of PCDA-PEG-Biotin and PCDA-PEG were measured to be 13,820 and 16,010 Dalton, respectively. It is worth noting that the PCDA-PEG-Biotin has a high CUR loading capacity of 27.0 wt.% as well as >500 fold greater water solubility than free CUR. We compared the stability of the PCDA-PEG-Biotin and CUR in phosphate buffered solution (PBS) at pH 7.4 by monitoring their UV absorption spectra (Figure S5A and C). It can be found that free CUR quickly degraded, leading to an about 55% loss within 30 min (Figure S5D), while no more than 5% of PCDA-PEG-Biotin decomposed after exposure for 24 h (Figure S5B). This indicates that the PCDA-PEG-Biotin indeed stabilizes CUR from decomposition as expected. Further, the DOX@PCDA-PEG-Biotin NPs were prepared by a facile O/W emulsion-solvent evaporation method without using any other emulsifiers or surfactants. The obtained nanoparticles were characterized by transmission electron microscopy (TEM), dynamic laser scattering (DLS) and zeta potential measurements. The TEM results show that the DOX@PCDA-PEG-Biotin NPs are spherical (Figure S6). The DOX@PCDA-PEG-Biotin NPs have a high DOX loading capacity of 11.9 wt.% with an encapsulation efficiency of 83.5%, possibly owing to the hydrophobic attraction, H-bonding and π‒π stacking interaction between CUR and DOX (Figure S1). The hydrodynamic diameter and zeta potential of the NPs are measured to be 82.1 nm and −9.70 mV, respectively (Table S2).

### GSH-triggered Degradation of Nano-carrier and Drug Release

**Figure 3:**
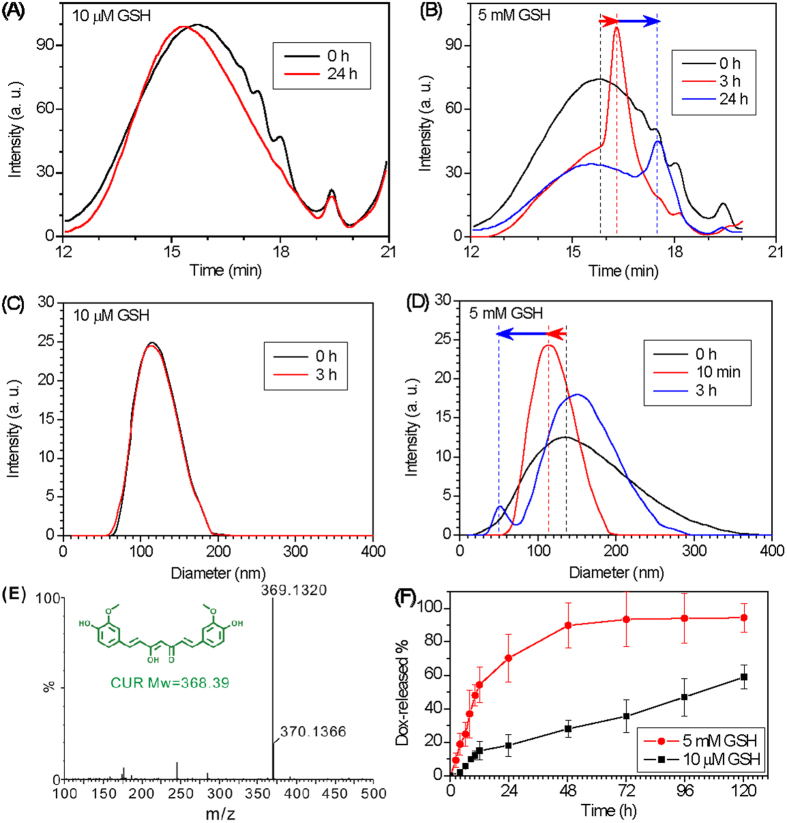
GSH-triggered degradation of the PCDA-PEG-Biotin nano-carrier (**A**‒**E**), and drug release behaviors of the DOX@PCDA-PEG-Biotin NPs (**F**). GPC (**A**,**B**) and DLS (**C**,**D**) profiles of the PCDA-PEG-Biotin in pH 7.4 PBS at 10 μM (**A**,**C**) and 5 mM GSH (**B**,**D**), respectively. (**E**) A LC-HR-MS spectrum showing the degraded species (CUR) and its assignment (M+H)+ peaks after treatment of the biotin-PEG-PCDA with 5 mM GSH and esterase (100 ) in PBS for 24 h. (**F**) DOX release profiles of the DOX@PCDA-PEG-Biotin NPs in pH 7.4 PBS at 10 μM and 5 mM GSH, respectively.

The intracellular GSH concentration generally ranges from 0.5 to 10 mM, whereas the extracellular concentration of GSH is remarkably lower (only 2–20 μM in plasma)^31^. We investigated the degradation of PCDA-PEG-Biotin NPs and the DOX release profiles of DOX@PCDA-PEG-Biotin NPs in PBS (pH 7.4) at two different concentrations of GSH (5 mM and 10 μM). As shown in Fig. 3, the molecular weight of PCDA-PEG-Biotin almost did not change after 24 h incubation with 10 μM GSH (Fig. 3A), but quickly reduced in the presence of 5 mM GSH (red and blue arrows in Fig. 3B). Correspondingly, the hydrodynamic diameter of the PCDA-PEG-Biotin nano-carrier remained almost unchanged at 10 μM GSH within 3 h, but rapidly reduced after incubation with 5 mM GSH (red arrow). These results demonstrate that the PCDA-PEG-Biotin NPs are stable at blood concentration of GSH, but tend to lose their integrity at an intracellular concentration of GSH. This degradation behavior is thought to be attributed to the GSH concentration-dependent cleavage of the disulfide bond on the PCDA backbone of PCDA-PEG-Biotin molecule. Furthermore, the LC-HR-MS spectrum displayed two characteristic peaks of (M+H)+ at about 369 and 370 m/z (Fig. 3E), revealing that the fragments degraded by 5 mM GSH were mainly CUR molecules (Mw = 368 Dalton). Therefore, it is expected that the PCDA-PEG-Biotin NPs will be stable for a considerably long period of time in the blood circulation, but will be readily degraded by high intracellular concentrations of GSH and then release the degraded fragment CUR once entering the targeted cancer cells^32^,^33^.

Meanwhile, the encapsulated DOX within the PCDA-PEG-Biotin NPs was also released responsively, as shown in Fig. 3F. In the absence of GSH, the DOX@PCDA-PEG-Biotin NPs almost did not release DOX. At a low concentration of GSH (10 μM), a sustained DOX release behavior of the DOX@PCDA-PEG-Biotin NPs was observed, while at a relatively high concentration of GSH (5 mM), the release of DOX from the DOX@PCDA-PEG-Biotin NPs became distinctly faster, owing to accelerated degradation and the disassembly of the hydrophobic PCDA zone as mentioned above.

### Reversal Effects on MDR in MCF-7/ADR Cells

**Figure 4:**
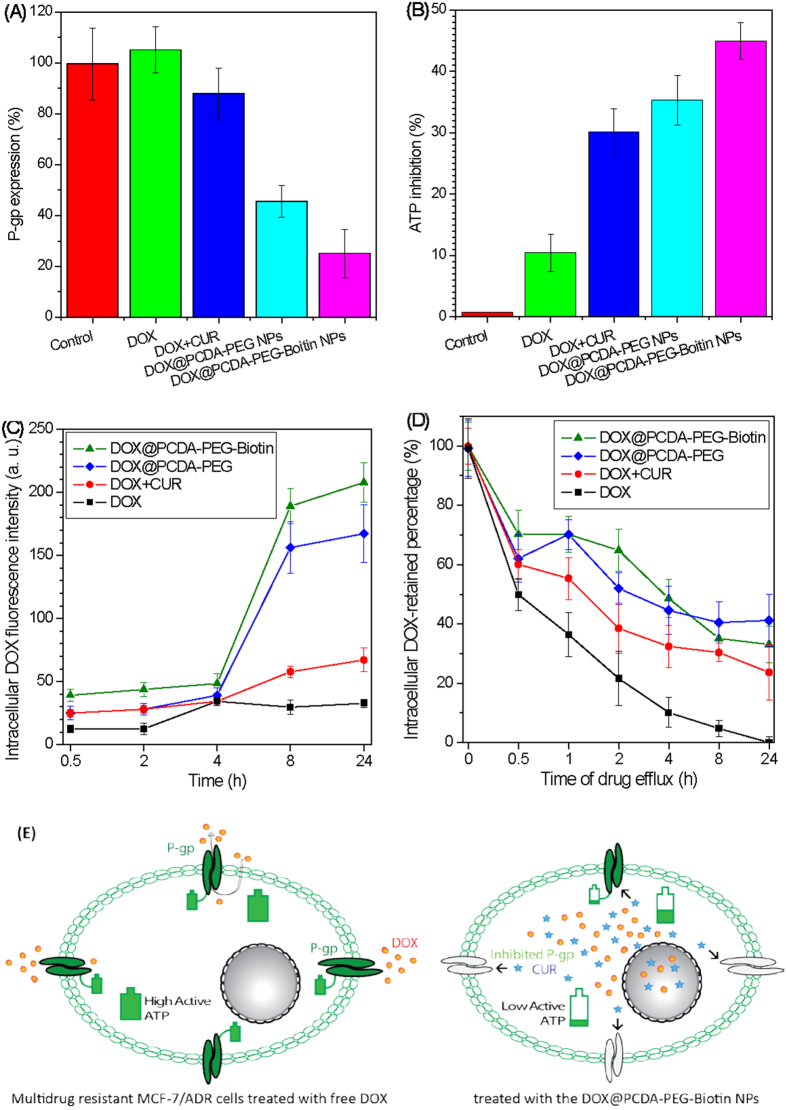
Reversal effects on MDR in MCF-7/ADR cells: the level of P-gp expression (**A**) and the ATP inhibition rate (**B**) on MCF-7/ADR cells after 48 h incubation with various formulas at an equivalent concentration of DOX; the accumulation of DOX fluorescence within MCF-7/ADR cells incubated for different duration of time (**C**); the retention percentage of intracellular DOX after removing extracellular DOX (**D**); schematic illustration of the reversal of MDR in MCR-7/ADR cells by the DOX@PCDA-PEG-Biotin NPs (**E**).

P-glycoprotein (P-gp), a product of the MDR1 gene, is a major adenosine triphosphate (ATP)-binding cassette (ABC) transporter which is able to function as an energy-dependent drug efflux pump and is linked to MDR in cancer cells^34^. ATP is a co-factor involved in drug efflux^35^. Herein, we investigated P-gp expression and ATP activity to evaluate reversal effects of the DOX@PCDA-PEG-Biotin NPs on MDR in MCF-7/ADR human breast carcinoma cells. Compared with blank control, free DOX inhibited ATP activity of MCF-7/ADR cells intensively (Fig. 4B) but also slightly increased P-gp overexpression (Fig. 4A), so that the intracellular accumulation of DOX was very limited (Fig. 4C,D), presumably due to a powerful MDR effect. Compared with free DOX, the combination of CUR with DOX not only inhibits ATP activity of MCF-7/ADR cells (Fig. 4B) but also suppresses P-gp expression (Fig. 4A), which leads to slightly increased intracellular accumulation and retention of DOX (Fig. 4C,D), confirming the chemosensitization of CUR. In contrast, the DOX@PCDA-PEG NPs which can co-deliver CUR and DOX inhibited the overexpression of P-gp more noticeably. Therefore, the intracellular accumulation and retention amounts of DOX were enhanced greatly (Fig. 4C,D). The DOX@PCDA-PEG-Biotin NPs further suppressed P-gp expression and APT activity, and improved the intracellular DOX accumulation and retention, presumably owing to the active targeting effect of conjugated biotin. Figure 4E schematically illustrates the reversal effects of the DOX@PCDA-PEG-Biotin NPs on MDR in MCF-7/ADR cells, involving reduction of P-gp expression, inhibition of ATP activity and increase of intracellular drug accumulation.

### Antitumor effects on MCF-7/ADR cells

We have determined the IC50 values (drug concentrations which reduce cell viability by 50%) of DOX and its formulations against multidrug resistant MCF-7/ADR cells in order to assess their *in vitro* antitumor effects. As indicated in Table 1, the IC50 values of DOX for MCF-7 and MCF-7/ADR cells were measured to be 0.21 μg/mL and 44.41 μg/mL, respectively. The drug resistance index (DRI, the ratio of IC_50_ values between MCF-7/ADR and MCF-7 cells) of MCF-7/ADR cells was calculated to be 211.48, indicating that cultured MCF-7/ADR cells were highly resistant to DOX. The cytotoxicities of CUR towards MCF-7 and MCF-7/ADR cells were relatively low as their IC_50_ values were determined to be 19.52 μg/mL and 51.76 μg/mL, respectively (Figure S6). However, DOX + CUR (0.8/1.0 (w/w)) had a much higher cytotoxicity against MCF-7/ADR cells (IC_50_ = 14.29 μg/mL) than DOX or CUR alone, owing to the chmosensitization of CUR as mentioned above. The reversal index (RI, the DRI of DOX /the DRI of DOX’s formulation) of DOX + CUR was calculated to be 3.10 (Table 1). The cytotoxicity of the PCDA-PEG-Biotin NPs against MCF-7 and MCF-7/ADR cells was dependent on their particle concentrations, but was still very limited at particle concentrations up to 160 μg/mL (Figure S8). However after combining/loading with DOX, the DOX@PCDA-PEG-Biotin NPs exhibited remarkably enhanced cytotoxicity against MCF-7/ADR cells as their IC_50_ value was considerably lowered to 1.88 μg/mL and their RI was as high as 23.62 (Table 1).

### *In vivo* tumor targeting and anti-MDR tumor efficacy

**Figure 5:**
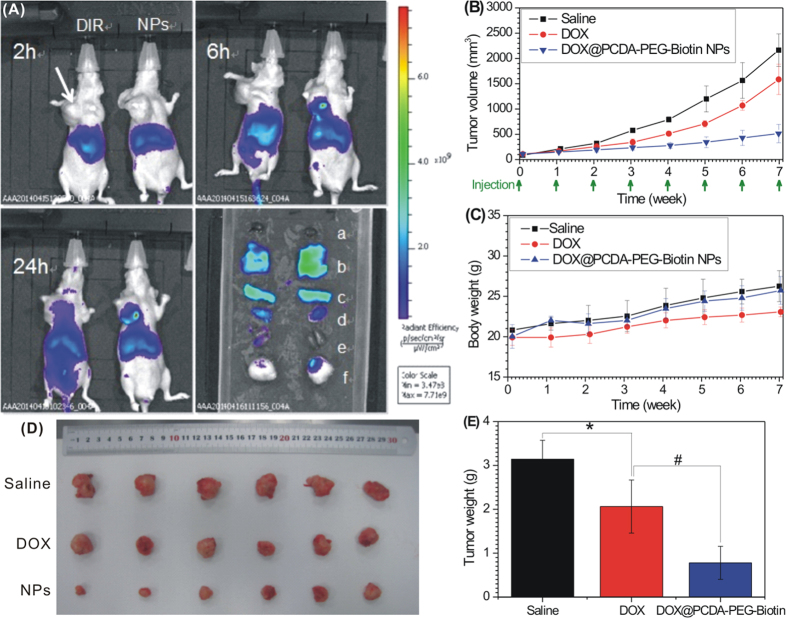
Tumor targeting capability and antitumor activity. (**A**) *In vivo* fluorescence images of the MCF-7/ADR tumor-bearing nude mice at 2, 6 and 24 h after intravenous injection of free DiR (left) and the DiR-labeled DOX@PCDA-PEG-Biotin NPs (right). The arrow indicates the sites of tumor. *Ex vivo* fluorescence Images of the tumor and normal tissues harvested from the euthanized MCF-7/ADR tumor-bearing nude mice at 24 h post injection, where a–f inset images represent heart, liver, spleen, lung, kidneys and tumor, respectively. The MCF-7/ADR tumor growth curves (**B**) and the body weight variation (**C**) after intravenous injection of free DOX, the DOX@PCDA-PEG-Biotin NPs at a DOX dose of 2 mg/kg each week, or saline with the equivalent volume (0.2 mL) as control. The appearances (**D**) and weights (**E**) of the tumors harvested from euthanized MCF-7/ADR tumor-bearing nude mice at 8 weeks. Significant differences: * (p < 0.01, compared to saline control); ^#^(p < 0.01, compared to free DOX).

To evaluate the tumor targeting capability of the DOX@PCDA-PEG-Biotin NPs, the *in vivo* biodistribution of 1,1′-dioctadecyl-3,3,3′,3′-tetramethylindotricarbocyanine iodide (DiR)-labeled DOX@PCDA-PEG-Biotin NPs intravenously administered into the MCF-7/ADR tumor-bearing nude mice was investigated by a non-invasive near-infrared (NIR) optical imaging technique. The DiR-labeled DOX@PCDA-PEG-Biotin NPs presented a stronger fluorescence signal in the tumor region over a short time (6 h) compared with free DiR (Fig. 5A). This means that the amount of DiR-labeled DOX@PCDA-PEG-Biotin NPs in the tumor was higher than that of free DiR. Compared with the normal tissues, stronger fluorescence signals were found at the tumor site within 24 h post injection of the DiR-labeled DOX@PCDA-PEG-Biotin NPs, reflecting a notable tumor targeting effect of the DOX@PCDA-PEG-Biotin NPs. At 24 h post injection, the mice were immediately euthanized, and several organs and tissues including tumor, heart, liver, spleen, lung and kidneys were harvested for *ex vivo* imaging. Compared with free DiR, the DiR-labeled DOX@PCDA-PEG-Biotin NPs had a much higher accumulation at the site of tumor site. These results clearly demonstrated an *in vivo* targeting ability of the DOX@PCDA-PEG-Biotin NPs.

Next, the antitumor effect of the DOX@PCDA-PEG-Biotin NPs was evaluated in MCF-7/ADR tumor xenograft model. As shown in Fig. 5, compared with free DOX, the DOX@PCDA-PEG-Biotin NPs presented a remarkably higher inhibition effect towards tumor growth, which is consistent with their *in vitro* antimultidrug resistant cancer effect. In addition, the body weights of the mice treated with DOX@PCDA-PEG-Biotin NPs, free DOX and saline had no significant change (Fig. 5C).

**Figure 6:**
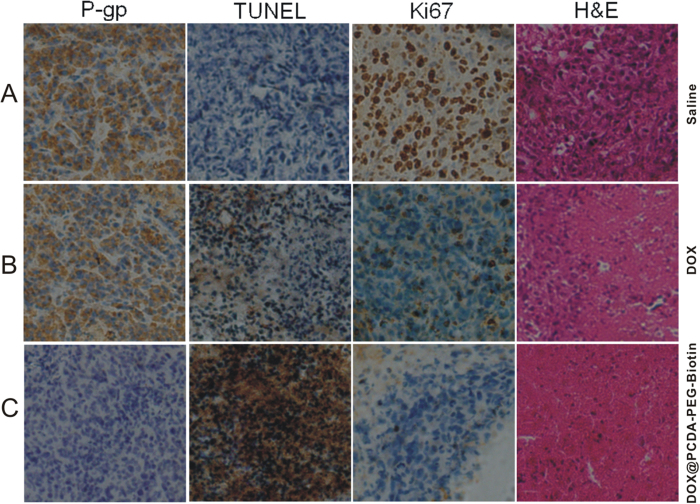
H&E, Ki67, TUNEL and P-gp images of tumor tissues dissected from the MCF-7/ADR tumor-bearing nude mice sacrificed at 8 weeks after intravenous injection of free DOX at a dose of 2 mg/kg each week (**B**), the DOX@PCDA-PEG-Biotin NPs at an equivalent DOX dose of 2 mg/kg each week (**C**), or an equivalent volume of saline as control **(A)**.

Histological examination by the hematoxylin and eosin (H&E) staining method showed that the treatment of the MCF-7/ADR tumor-bearing nude mice with the DOX@PCDA-PEG-Biotin NPs led to a great reduction in cancer cell density in the tumor tissue (Fig. 6). Moreover, the *in situ* Ki67 and TUNEL assays indicated that the treatment with DOX@PCDA-PEG-Biotin NPs significantly inhibited cancer cell proliferation and induced apoptosis in the tumor tissue (Fig. 6). Furthermore, the *in situ* P-gp assays showed that DOX@PCDA-PEG-Biotin NPs had greatest inhibition to P-gp expression in the tumor tissue as compared to other groups. Collectively, these results confirmed that the DOX@PCDA-PEG-Biotin NPs efficiently accumulated at the tumor site and thereby achieved an optimal antitumor efficacy *in vivo*.

## Conclusion

In summary, we have developed a self-assembled polycurcumin nanoparticle, which can be used as both a pre-chemosensitizer and a nano-carrier of hydrophobic anticancer drug, by incorporating CUR into an intracellular cleavable polymer backbone. This nanoparticle shows GSH levels-dependent degradation and drug co-release properties, which are selectively degraded into CUR and simultaneously release the encapsulated DOX at intracellular GSH levels for enhanced biological specificity and therapeutic efficacy of multidrug resistant cancer. The hydrophilic outer shell of biotin-PEG offers effective protection against recognition and uptake by the reticuloendothelial system, allowing for stealth-shielding and long circulation, as well as biotin receptor-mediated tumor targeting. Furthermore, the polycurcumin nanoparticles could effectively reverse DOX resistance through suppressing P-gp expression and ATP activity, leading to enhanced cellular uptake of DOX and the reduced drug efflux in multidrug resistant MCF-7/ADR cells. Here DOX was incorporated into the hydrophobic polycurcumin inner core; we expected that other hydrophobic drugs could also be loaded into the polycurcumin domain through their hydrophobic interaction and be released by intracellular level of GSH triggered polycurcumin degradation.

## Experimental Section

### Materials

3, 3′-dithiodipropionic acid, N, N′-dicyclohexylcarbodiimide, 4-dimethylaminopyridine, glutathione, methoxyl poly (ethylene glycol) (Mw = 5000 Dalton), poly (ethylene glycol) (Mw = 6000 Dalton) and biotin were purchased from Aladdin chemistry Co., Ltd. (Shanghai, China). Curcumin and doxorubicin hydrochloride were purchased from Alfa-Aesar. 3-(4,5-dimethylthiazol-2-yl)-2,5-diphenyl-tetrazolium bromide and dimethyl sulfoxide were purchased from Solarbio science & technology (Beijing, China). Propidium iodide was purchased from Solarbio science technology (Beijing, China).

### Synthesis and characterization of biotin-PEG-PCDA and PEG-PCDA

PCDA: CUR (1.000 g), 3, 3′-dithiodipropionic acid (0.571 g), N, N′-dicyclohexylcarbodiimide (DCC) (1.150 g) and 4-dimethylaminopyridine (DMPA) (0.100 g) were dissolved in 40 mL anhydrous dichloromethane and were magnetically stirred at room temperature for 24 h. The resultant reaction solution was firstly filtrated to remove the formed precipitate and then added into an excess of anhydrous ether to make the crude PCDA product precipitate. The PCDA product was purified by dichloromethane dissolution, anhydrous ether precipitation and filtration for three times and finally dried under vacuum (PCDA, 0.95 g, yield ~60%). PCDA-PEG: PCDA (0.90 g), methoxyl poly (ethylene glycol) (1.125 g), DCC (46 mg) and DMAP (2.74 mg) were dissolved in 40 mL anhydrous dichloromethane and magnetically stirred at room temperature for 24 h. The resultant reaction solution was filtrated and added into an excess of anhydrous ether to make the crude PCDA-PEG product precipitate. The PCDA-PEG was purified by dialyzing the crude PCDA-PEG in against deionized water for 24 h with a dialysis membrane (Mw cut-off of 6000 Dalton). The pure PCDA-PEG was obtained by lyophilization and kept under dry conditions (1.76 g, yield ~87%). PCDA-PEG-Biotin: biotin (0.066 g), poly (ethylene glycol) (1.350 g), DCC (0.111 g), DMAP (9.9 mg) were dissolved in 40 mL anhydrous dichloromethane and magnetically stirred at room temperature for 24 h. After filtration to remove the formed insoluble by-product, the filtrate was added into an excess of anhydrous ether to make the formed PEG-Biotin precipitate. The pure PEG-Biotin was obtained through multiple precipitations from dichloromethane with anhydrous ether and dried under vacuum (1.27 g, yield ~90%). PCDA (0.90 g), PEG-Biotin (0.337 g), DCC (22 mg) and DMAP (2 mg) were dissolved in 40 mL anhydrous dichloromethane and stirred at room temperature for 24 h. After filtration to remove the insoluble by-product, the filtrate was added into an excess of anhydrous ether to make the formed PCDA-PEG-Biotin precipitate. The pure PCDA-PEG-Biotin was obtained by dialyzing against deionized water for 24 h with a dialysis membrane (Mw cut-off of 6000 Dalton) and then lyophilization (1.03 g, yield ~%). The synthesized products were characterized by 1 H-NMR on a Varian- Mercury Plus (400 MHz) spectrometer with CDCl3 as solvent and tetramethylsilane as an internal standard and GPC on a Waters HPLC system equipped with a model 1525 binary HPLC pump, a model 2414 refractive index detector and a series of Styragelr-Rcolumns (HR3 and HR4) (tetrahydrofuran was used as an eluent at a flow rate of 1.0 mL/min and the MWs were calibrated with polystyrene standard).

### Solubility measurements and stability tests

The water solubility of CUR, PCDA-PEG and PCDA-PEG-Biotin was determined by UV. The saturated aqueous solution of CUR, PCDA-PEG and PCDA-PEG-Biotin was diluted with DMSO and quantitatively by UV at 400 nm (PCDA-PEG and PCDA-PEG-Biotin) or 420 nm (CUR) on a HITACHI U-2910 spectrophotometer. The stability of CUR, PCDA-PEG and PCDA-PEG-Biotin in aqueous media at physiological pH of 7.4 was tested by monitoring their UV-visible absorption spectra.

### Preparation and characterization of DOX@PCDA-PEG and DOX@PCDA-PEG-Biotin NPs

Doxorubicin HCl was converted to its hydrophobic form (DOX) by neutralizing the hydrochloride salt with the base triethylamine. The DOX@PCDA-PEG and DOX@PCDA-PEG-Biotin NPs were prepared by using an emulsion/solvent evaporation method. For example, a solution containing 10 mg of PCDA-PEG-Biotin or PCDA-PEG, 2 mg of DOX in 3 mL of 12.5% (v/v) methanol in chloroform solution (12.5:87.5) was emulsified in10 mL of aqueous solution to form an oil-in water emulsion. The emulsification was carried by using a probe-type sonicator (Soniprep 150, Sanyo) under an ice bath at 200 w for 5 min. Afterwards, the organic solvent was evaporated by rotary vacuum at 40 °C and the resulting suspension was centrifuged at 4000 rpm for 20 min to remove any aggregated particles and the unencapsulated free DOX. The obtained clear supernatant was then lyophilized to obtain the DOX@PCDA-PEG and DOX@PCDA-PEG-Biotin NPs. The drug free NPs were prepared by using a similar procedure without adding DOX. The particle size and zeta potential were measured by the Zetasizer (Nano ZS, Malvern). For TEM characterization, a drop of diluted solution of PCDA-PEG or PCDA-PEG-Biotin NPs was dropped onto a TEM copper grid (300 mesh) and then stained with 1% (w:v) phosphotungstic acids and dried before measurement. The sample was observed by TEM (JEM-2010/INCA OXFORD). The drug encapsulation efficiency and the drug loading was determined by using a F-7000 fluorescence spectrophotometer (the excitation wavelength set at 497 nm and the emission wavelength set at 555 nm).

### GSH triggered degradation

10 mg PCDA-PEG-Biotin was dissolved in 10 mL phosphate buffered solution (PBS, pH 7.4) containing 10 μM or 5 mM GSH and stirred at 37 °C. At certain time intervals, aliquots of the solution were withdrawn for DLS measurement and then lyophilized. The lyophilized product was dissolved in tetrahydrofuran for GPC measurement.

### LC-HRMS Analysis

LC-HRMS was performed on a Waters ACQUITY UPLC system equipped with a binary solvent delivery manager and a sample manager, coupled with a Waters Micromass Q-TOF Premier Mass Spectrometer equipped with an electrospray interface (Waters Corporation, Milford, MA).

### *In vitro* GSH-triggered DOX release

The DOX release tests were carried out by a dialysis method. For example, DOX@PCDA-PEG-Biotin NPs (0.5 mL) were added into a dialysis bag with a molecular weight cut-off of 3500 Da) against 5 mL of the phosphate buffer solution (pH 7.4, 1% Tween-80) containing different concentrations of GSH, and gently shaken at 37 °C in a shaker at 120 rpm. At predetermined time intervals, the total buffer solution was withdrawn, followed by replacing with 5 ml of fresh buffer solution with the same GSH concentration. The fluorescence intensity of Dox was measured by a F-7000 fluorescence spectrophotometer.

### Cell culture

MCF-7 cells were obtained from the American Type Culture Collection (ATCC, manassas, VA). MCF-7/ADR cells were kindly donated from Prof. Shen Qi group, School of Pharmacy, Shanghai Jiaotong University (China). MCF-7 cells were culture in RPMI 1640 medicum containing 10% fetal bovine serum, 100 U/mL penicillin G sodium and 100 μg/mL streptomycin sulfate (complete 1640 medium) MCF7/ADR cells were grown in complete 1640 medium with 1 μg/mL doxorubicin (DOX). Cells were maintained at 37 °C in a humidified and 5% CO2 incubator.

### *In vitro* cytotoxicity

MCF-7 or MCF-7/ADR cells (5 × 103 cells per well) were seeded in 96-well plates. After culture for 24 h, the cells were exposed to the DOX solution and the DOX-loaded NPs with different concentrations of DOX for 48 h, followed by adding 0.1 ml MTT (0.5 mg/ml). After 4 h of incubation, the culture medium was then removed and the cells were mixed with 0.1 ml of dimethyl sulphoxide. The absorbance was measured at a test wavelength of 570 nm by an ELISA plate reader (Varioskan Flash).

### Flow cytometry

The fluorescence intensity of DOX in cells was measured by flow cytometry (FACS Calibur, BD, USA) and analyzed with CellQuest software through fluorescence channel2 (FL2).

### P-gp and ATP assays

P-gp assay: MCF-7/ADR cells (2 × 105 cell/well) were seeded in 6-well plates. After culture for 24 h, the cells were exposed to the DOX solution (5 μg/mL) and the DOX solution with 5 μg/mL DOX and a CUR or its equivalent concentration of 6 μg/mL for 48 h at 37 °C. Then the cells were trypsinized, collected, and resuspended in 0.1 mL of PBS (pH 7.4) for flow cytometry analysis. PE-conjugated mouse anti-human monoclonal antibody against P-gp was use to label cells according to the manufacture’s instruction, and the nonspecific labeling was corrected by its isotype control. ATP assay: MCF-7/ADR cells (5 × 104 cells/well) were seeded in 24-well plates. After culturing for 24 h, the cells were exposed to the DOX solution (5 μg/mL) and the DOX solution with 5 μg/mL DOX and a CUR or its equivalent concentration of 6 μg/mL for 48 h at 37 °C. Then the cells were washed with ice-cold PBS three times and lysed with ATP lysis buffer for ATP assay. The intracellular ATP levels were determined using a luciferin/luciferase assay according to the protocol of the ATP assay kit (Beyotime,China).

### Animals and tumor xenograft models

All animals received care in compliance with the guidelines outlined in the Guide for the Care and Use of Laboratory Animals and all procedures were approved by the Animal Care and Use Committee of Shanghai Jiao Tong University. To set up the tumor xenograft model, the female BALB/C nude mice (6 weeks, 18 ~ 22 g) were subcutaneously inoculated in the right axilla with 6 × 10^7^ MCF-7/ADR cells. The tumor size was monitored by a vernier calliper and the tumor volume (V) was calculated as V = L × W2/2, where L and W were the length and width of the tumor, respectively.

### *In vivo* imaging study

When the tumors reached to ~500 mm^3^, the mice were intravenously injected by DiR-labeled PCDA-PEG-Biotin NPs and free DiR (10 μg), respectively. Images were taken on the IVIS-RLumina II imaging system (Caliper, USA, excitation: 748 nm, emmision:780 nm) at 2, 6 and 24 h post injection. After the 24 h scanning, the mice were euthanized. The tumors as well as major organs were harvested and subjected for *ex vivo* imaging.

### *In vivo* antitumor efficacy

The tumor-bearing mice were weighed and randomly divided into different groups when the tumor volume reached to 100 mm^3^. From Day 0, the mice were intravenously injected with DOX solution (2 mg/kg), DOX@PCDA-PEG-Biotin NPs (2 mg/kg) and saline as a negative control once a week for 8 weeks, and meanwhile the body weight and the tumor size was measured. At Day 2 after the last drug administration, the mice were euthanized, and the tumor as well as the organs (heart, liver, spleen, lung, kidney) were collected and, weighed, washed with saline thrice and fixed in the 10% neutral-buffered formalin. For the hematoxylin and eosin staining, the formalin-fixed tumors were embedded in paraffin blocks and visualized by Olympus BX 51 microscope. For the TUNEL apoptosis staining, the fixed tumor sections were stained by the *in Situ* Cell Death Detection Kit (Roche Applied Science) according to the manufacturer protocol. The Ki-67 staining was conducted by using the labeled streptavidin-biotin method.

The stained tumor slides were observed by Olympus BX 51 microscope. The inhibition rate of tumor growth (IRT) was calculated according to the following formula: IRT = 100% × (mean tumor weight of a control group − mean tumor weight of a treatment group)/mean tumor weight of a control group. The IRT values are listed in Table S3.

## Additional Information

**How to cite this article**: Guo, S. *et al*. A nanoparticulate pre-chemosensitizer for efficacious chemotherapy of multidrug resistant breast cancer. *Sci. Rep.* **6**, 21459; doi: 10.1038/srep21459 (2016).

## Supplementary Material

Supplementary Information

## Figures and Tables

**Figure 1:**
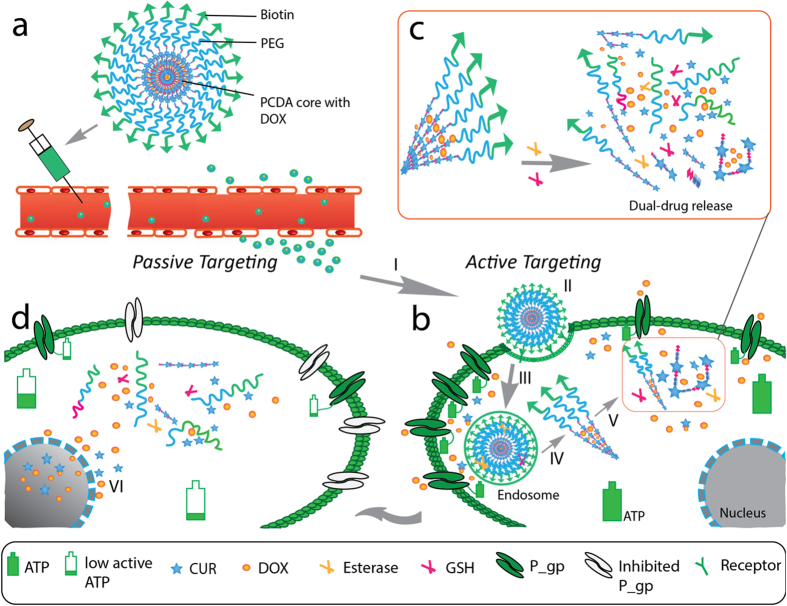
Schematic illustration of the treatment of multidrug-resistant cancer with the DOX@PCDA-PEG-Biotin NPs. After intravenous injection, the DOX@PCDA-PEG-Biotin NPs accumulate at the site of tumor through passive targeting (EPR effect) (**a**), and then are taken up by MDR cancer cells through active targeting (biotin acceptor-mediated endocytosis) (**b**). Subsequently, the DOX@PCDA-PEG-Biotin NPs are liable to escape from endosome/lysosome and then enter cytosol, where they will encounter relatively high level of GSH and thus decomposing into CUR followed by the release of encapsulated DOX (**c**). The released CUR can down-regulate the P-gp expression on MDR cancer cells and inhibit their ATP activity in favor of the intracellular and intranuclear accumulation of released DOX and CUR (**d**), playing a role as pre-chemosensitizer.

**Table 1:** The IC_50_, DRI and RI values of various formulas against MCF-7/ADR cells.

Treatment^c^	IC_50_ value (μg/mL)^a^	DRI^d^	RI^e^
DOX	44.41	211.48	
DOX+CUR^b^	14.29	68.05	3.10
DOX@PCDA-PEG NPs	3.48	16.58	12.70
DOX@PCDA-PEG-Biotin	1.88	8.95	23.62

^a^The IC_50_ value of DOX against MCF-7 cells was measured to be 0.21 μg/mL; ^b^DOX+CUR (DOX/CUR = 0.8: 1.0 wt./wt.); ^c^all formulas contain an equivalent amount of DOX; ^d^the drug resistance index (DRI) of MCF-7/ADR cells was calculated as the IC_50_ for MCF-7/ADR cells/the IC_50_ for MCF-7 cells; ^e^the reversal index (RI) was calculated as the DRI of DOX/the DRI of the formulation containing DOX.
