# Supplementary material for: A nanoparticulate pre-chemosensitizer for efficacious chemotherapy of multidrug resistant breast cancer
Source: Sci Rep. 2016 Feb 15;6:21459. doi: 10.1038/srep21459 (PMC4753478; doi:10.1038/srep21459)
Supplement: Supplementary Information [file srep21459-s1.doc]

**Supporting Information**

A nanoparticulate pre-chemosensitizer for efficacious chemotherapy of multidrug resistant breast cancer

Shengrong Guo^1^, Li Lv^1^, Yuanyuan Shen^1^, Zhongliang Hu^2^, Qianjun He^3^, and Xiaoyuan Chen^4^

^1^School of Pharmacy, Shanghai Jiao Tong University, Shanghai 200240, China. ^2^School of Chemical and Process Engineering, University of Leeds, Leeds LS3 9BD, UK. ^3^Guangdong Key Laboratory for Biomedical Measurements and Ultrasound Imaging, Department of Biomedical Engineering, School of Medicine, Shenzhen University. ^4^Laboratory of Molecular Imaging and Nanomedicine (LOMIN), National Institute of Biomedical Imaging and Bioengineering (NIBIB), National Institutes of Health (NIH), Bethesda MD 20892, USA. Correspondence and requests for materials should be addressed to Prof. Dr. S. Guo (email:srguo@sjtu.edu.cn), Prof. Dr. Q. He (email: nanoflower@126.com ) or Prof. Dr. X. Chen (email: [shawn.chen@nih.gov](mailto:shawn.chen@nih.gov))


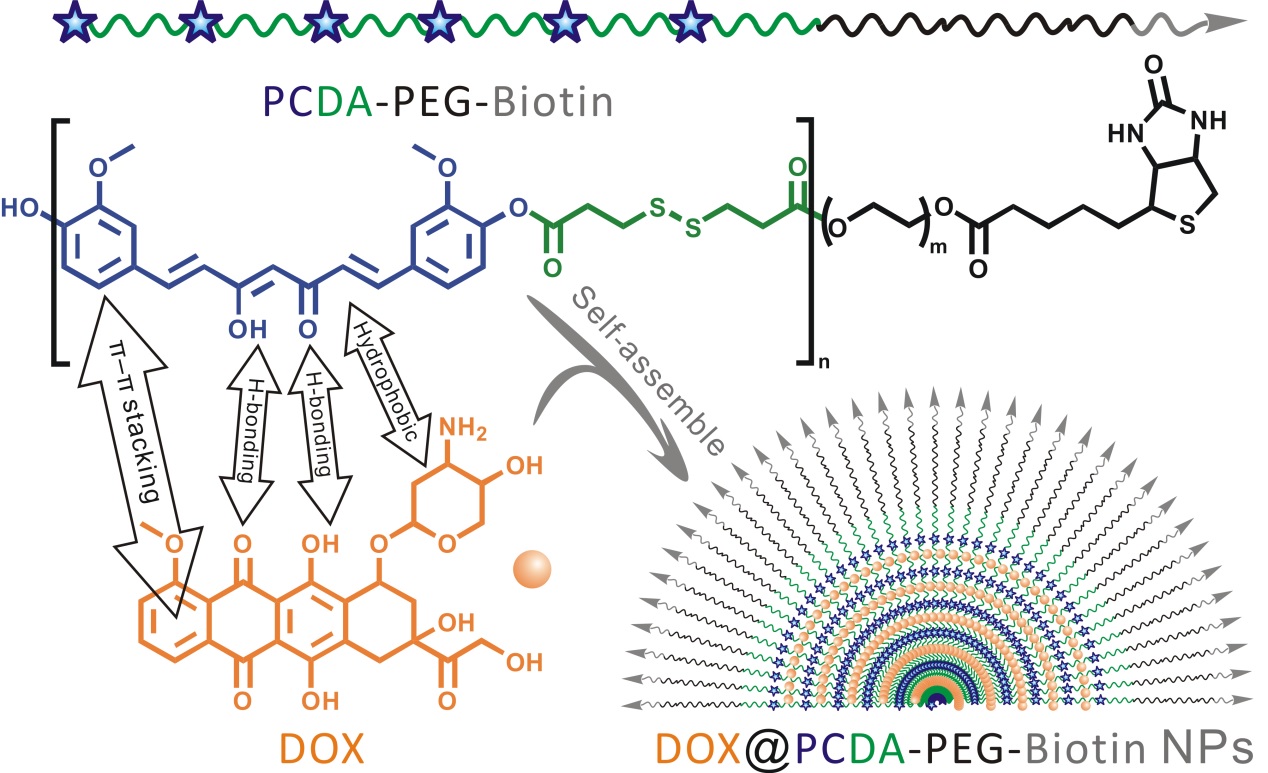


**Figure S1.** Self-assembly of the DOX@PCDA-PEG-Biotin NPs via several attractions including the π‒π stacking, the H-bonding and the hydrophobic interaction.


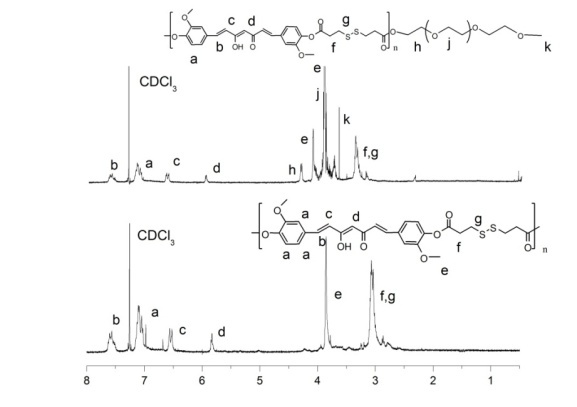


**Figure S2.** ^1^H NMR spectra of PCDA and PCDA-PEG.


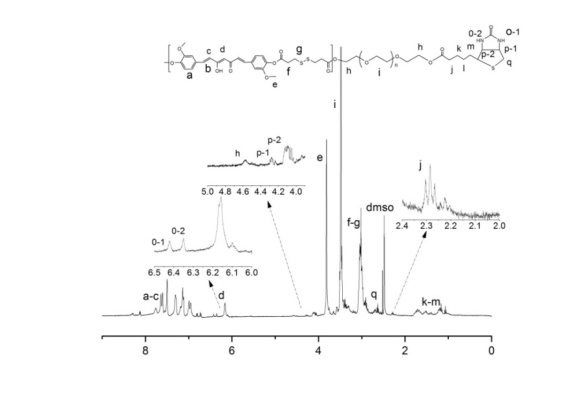


**Figure S3.** ^1^H NMR spectrum of PCDA-PEG-Biotin.


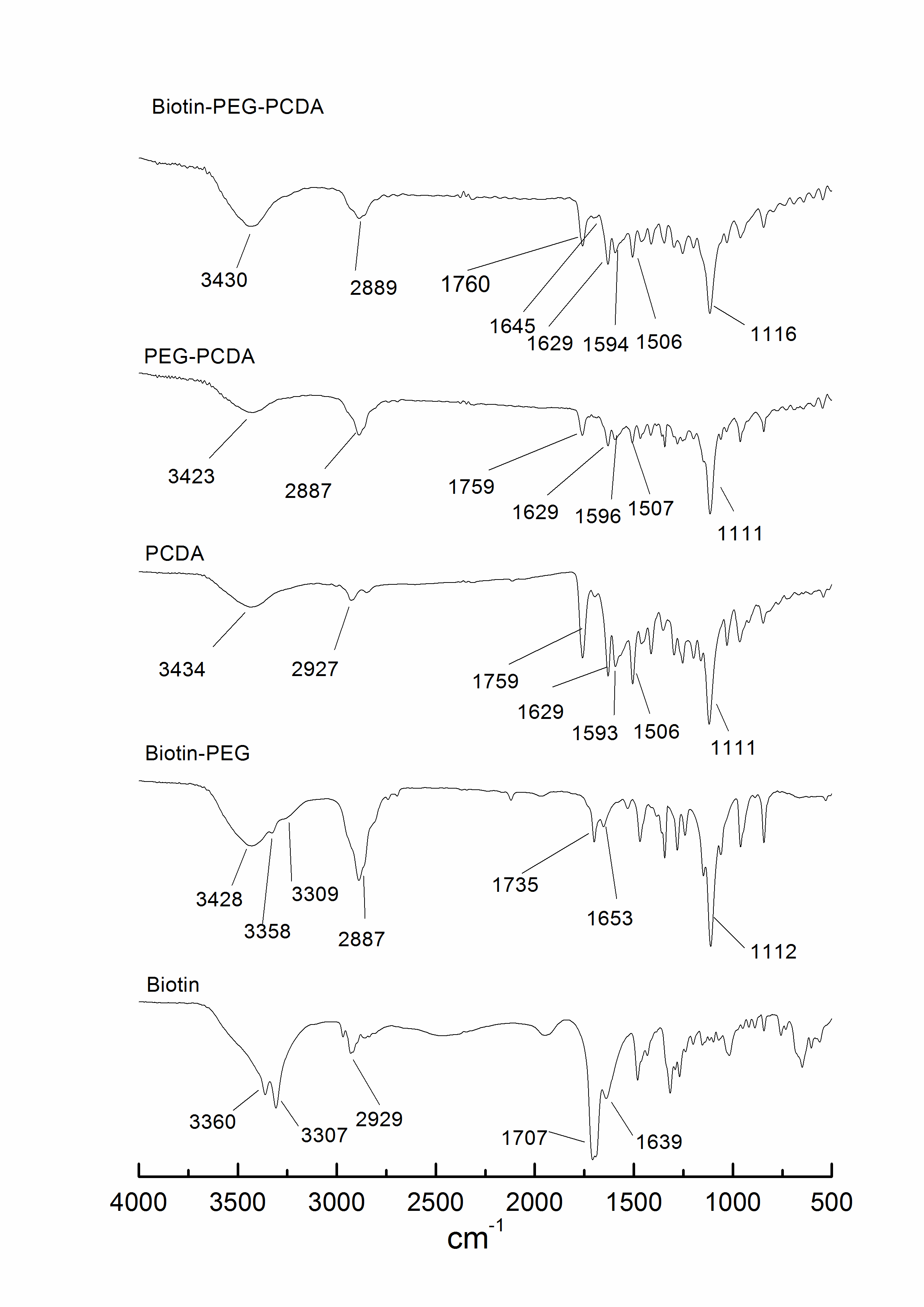


**Figure S4.** FT-IR spectra of PCDA, PCD-PEG and PCDA-PEG-Biotin.

**Figure S5.** The UV absorption spectra of PCDA-PEG-Biotin (A) and CUR (B) after immersion in pH 7.4 PBS for different duration of time, and time-dependent degradation percentages of PCDA-PEG-Biotin (C) and CUR (D).


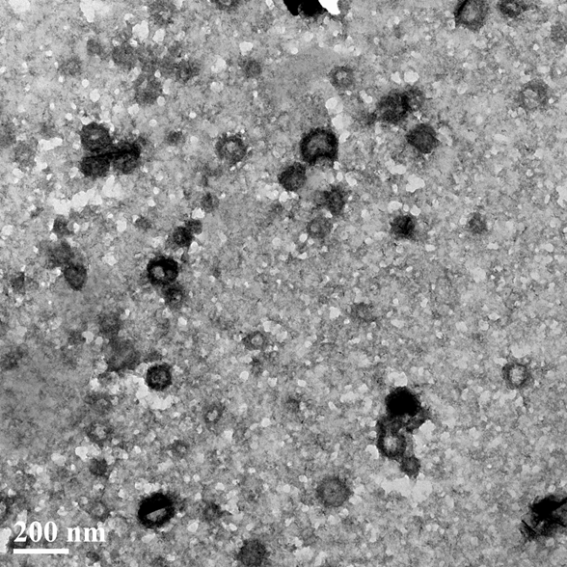


**Figure S6.** TEM image of the DOX@PCDA-PEG-Biotin NPs.

A


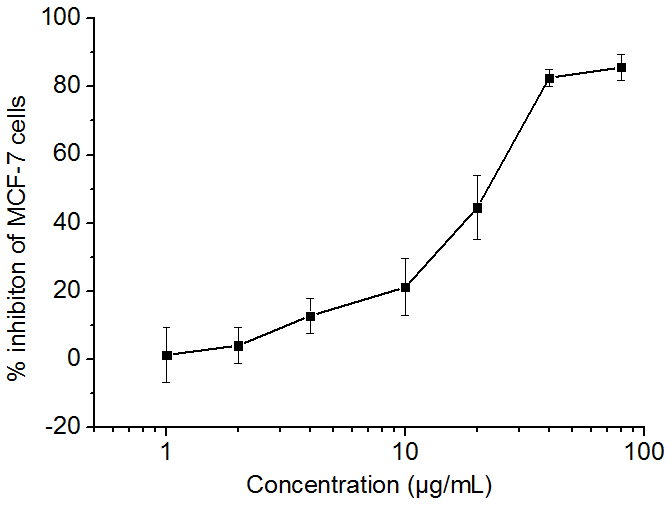


B


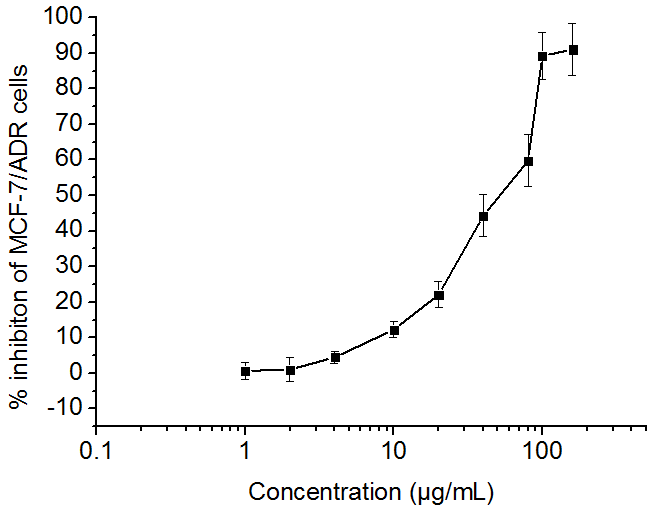


**Figure S7.** Inhibition rates of MCF-7 (A) and MCF-7/ADR (B) cells after incubation with CUR. The IC50 values for MCF-7 and MCF-7/ADR cells are measured to be 19.52 μg/mL and 51.76 μg/mL, respectively.

A

B

**Figure S8.** Viability of MCF-7(A) and MCF-7/ADR (B) after 48 h incubation with PCDA-PEG-Biotin. The viabilities of MCF-7 or MCF-7/ADR cells still maintain above 70% when the CUR equivalent concentration of PCDA-PEG-Biotin is up to 160 μg/mL.

**Table S1.** Characterization of PCDA-PEG-Biotin and PCDA-PEG.

|  | Mw (Dalton) | PDI | Solubility in water (μg/mL) |
| --- | --- | --- | --- |
| PCDA | 10480 | 1.90 | — |
| PCDA-PEG | 13820 | 1.55 | 360 (84*) |
| PCDA-PEG-Biotin | 16010 | 1.73 | 470 (127*) |
| CUR | — | — | 0.25 |

* Concentrations of equivalent CUR

**Table S2.** Biophysical parameters of the DOX@PCDA-PEG-Biotin NPs (*n* = 3).

| Nanoparticles | DOX loading capacity (%) | Encapsulation efficiency (%) | Paritcle size (nm) | PDI | Zeta potential (mV) |
| --- | --- | --- | --- | --- | --- |
| PCDA-PEG NPs | — | — | 75.2±1.0 | 0.11±0.13 | -0.6±0.4 |
| DOX@PCDA-PEG NPs | 12.7±0.8 | 87.0±1.6 | 79.5±1.9 | 0.16±0.01 | -0.5±1.7 |
| PCDA-PEG-Biotin NPs | — | — | 76.5±1.6 | 0.13±0.02 | -0.08±1.5 |
| DOX@PCDA-PEG-Biotin NPs | 11.9±1.192 | 83.5±1.7 | 82.1±2.0 | 0.13±0.01 | -0.7±0.7 |

**Table S3.** Body weights of mice and inhibitory rates of tumor (IRT) after injection of saline, free DOX, and the DOX@PCDA-PEG-Biotin NPs at a weekly equivalent DOX dose of 2 mg/kg for 8 consecutive weeks.

|  | Tumor weight (g) | IRT (%) |
| --- | --- | --- |
| Saline  Free DOX  DOX@PCDA-PEG-Biotin | 3.14±0.43  2.06±0.60  0.77±0.38 | —  34  75 |
